# MaPac2, a Transcriptional Regulator, Is Involved in Conidiation, Stress Tolerances and Pathogenicity in *Metarhizium acridum*

**DOI:** 10.3390/jof11020100

**Published:** 2025-01-28

**Authors:** Xiaobin Hu, Baicheng Li, Yan Li, Yuxian Xia, Kai Jin

**Affiliations:** 1 Genetic Engineering Research Center, School of Life Sciences, Chongqing University, Chongqing 401331, China; 202226021021@cqu.edu.cn (X.H.); 201826021014@cqu.edu.cn (B.L.); 202426021010@cqu.edu.cn (Y.L.); 2 Chongqing Engineering Research Center for Fungal Insecticide, Chongqing 401331, China; 3 Key Laboratory of Gene Function and Regulation Technologies, Chongqing Municipal Education Commission, Chongqing 401331, China; 4 National Engineering Research Center of Microbial Pesticides, Chongqing 401331, China

**Keywords:** entomopathogenic fungus, *Metarhizium acridum*, *MaPac2*, stress tolerances, pathogenicity

## Abstract

The Gti1/Pac2 protein family, which is highly conserved across fungi, is pivotal in processes such as fungal development, spore formation, protein export, toxin production, and virulence. Despite its importance, the precise functions of *Pac2* within entomopathogenic fungi have yet to be fully understood. In our study, the *MaPac2* gene from *M. acridum* was identified, and its functions were explored. Studying the domain of the protein showed that MaPac2 comprises 422 amino acids with a characteristic Gti1/Pac2 family domain (Pfam09729). Additionally, MaPac2 is predicted to have an N-terminal protein kinase A phosphorylation site and a potential cyclin-dependent kinase phosphorylation site, highlighting its potential regulatory roles in the fungus. Our findings indicate that the inactivation of *MaPac2* resulted in faster germination of conidia and a marked reduction in conidial production. Furthermore, stress tolerance tests revealed that the absence of *MaPac2* significantly bolstered the fungal resilience to UV-B radiation, heat shock, SDS exposure, and stresses induced by hyperosmotic conditions and oxidative challenges. Virulence assessments through bioassays indicated no substantial differences among the WT, *MaPac2*-disrupted strain, and CP strains in the topical inoculation trials. Interestingly, deletion of *MaPac2* increased the fungal virulence by intrahemocoel injection. Furthermore, we found that disruption of *MaPac2* impaired fungal cuticle penetration due to the diminished appressorium formation but increased the fungal growth in locust hemolymph. These findings provide further insights into the roles played by Gti1/Pac2 in insect pathogenic fungi.

## 1. Introduction

The Gti1/Pac2 protein family, sometimes referred to as the Wor1, Pac2, and Ryp1 (WOPR) families, is highly conserved in fungi and generally consists of two proteins, Gti1 and Pac2 [1]. A defining characteristic of the Gti1/Pac2 protein family is their N-terminal conserved domain, known as the WOPR box. This domain is responsible for binding to particular DNA sequences within the promoter region, thereby controlling the transcription process [1,2]. The WOPR box is composed of two highly conserved domains, WOPRa and WOPRb, which are structurally intertwined and cannot bind to DNA independently [3,4]. They form a compact globular domain separated by a variable-length, less conserved “linker” region that varies in length across different species [3,5]. Compared to the N-terminal region, there is a notable diversity in the C-terminal region among proteins of the Gti1/Pac2 family across different species [6].

Thus far, the Gti1/Pac2 protein family has been well studied in human and plant pathogenic fungi and plays critical roles in fungal growth, sporulation, protein secretion, toxin synthesis, and pathogenicity. In existing species research, Gti1/Pac2 proteins occur in pairs and control different processes [7]. Among them, the Pac2 protein controls various biological processes in existing species studies. Regarding the utilization of nutrients, the proteins Pac2 from *Schizosaccharo-myces pombe* are involved in adapting to changes in nutrition [8]. Specifically, Pac2 regulates the commencement of sexual development under varying nitrogen availability [8]. Regarding hyphal growth and development, the *Pac2* homolog *YHR177W* in *Saccharomyces cerevisiae* exerts no influence on morphological transitions [9]. *MoPac2* (*Pac2* homolog) from *Magnaporthe oryzae* is crucial for fungal development. Although it is dispensable for the formation of perithecia and ascospores, its deletion results in approximately a twofold enhancement in conidiation [10]. In the context of chemical sensitivity, although *Gti1* exhibits variable responses to distinct stress conditions, the impact of *Pac2* homologs on stress responses remains inconclusive based on the limited studies conducted [1,10,11,12]. With respect to osmotic and invasive growth, Pac2 affects mycelial invasive growth, and Δ*MoPac2* mutants display hyphal growth defects [13]. *Gti1* is pivotal for pathogenicity, while *Pac2* is essential for full virulence in *M. oryzae* [10]. The deletion of *Fgp2*, a *Pac2* homolog, does not impact toxin synthesis in *Fusarium graminearum* [14]. Furthermore, the elimination of *FoPac2* or *MoPac2*, both *Pac2* homologs, significantly diminishes fungal virulence, while the absence of *Fgp2* in *F. graminearum* does not affect its pathogenic capabilities [10,15]. Current studies suggest that the functions of *Pac2* are highly variable among different fungal species. Currently, the functional studies of the Gti1/Pac2 protein family predominantly concentrate on Gti1 and its analogous genes, while research on *Pac2* is relatively limited. At the same time, the functions of *Pac2* in insect pathogenic fungi are currently unclear.

Pest management is a pivotal element in safeguarding agricultural growth and ensuring food security [16]. While chemical pesticides have been instrumental in boosting agricultural yields, their use has concurrently resulted in a range of environmental challenges and fostered the evolution of resistance among pests [17]. From the year 2000 onwards, there has been a surge in the global emphasis on environmental conservation and heightened consciousness regarding food safety [18]. In response, biopesticides, known for their eco-friendly properties and precise targeting, have garnered widespread acceptance [19]. As crucial biocontrol agents, entomopathogenic fungi undergo a three-stage pathogenic process: initially, they adhere to the insect exoskeleton, and then, they breach the cuticle, followed by the colonization of the hemocoel, ultimately leading to the host death [19,20]. Entomopathogenic fungi possess a broad range of potential applications due to their environmentally benign nature, their reduced likelihood of triggering resistance in hosts, and their ability to provide sustainable pest management solutions [21]. Nevertheless, there are constraints to employing entomopathogenic fungi in pest control. One such constraint is the extended period it takes for these fungi to eliminate their insect targets, which can range from 6 to 12 days [22]. Additionally, the extensive application of these fungi is hindered by high manufacturing expenses and their vulnerability to environmental influences [23,24]. Consequently, delving into the molecular mechanisms underlying the infection process and thoroughly investigating the biocontrol capabilities of entomopathogenic fungi hold significant theoretical and practical implications.

The entomopathogenic fungus *Metarhizium acridum*, widely recognized, has proven effective in the management and control of locust populations [25]. To date, *M. acridum* has become a model fungus for investigating pathogen‒host interactions and make important contributions to screening and identifying genes, which play crucial roles in conidiation, stress tolerance, and pathogenicity [26]. In this study, the gene *MaPac2* in *M. acridum* was pinpointed, and its functions were thoroughly examined.

## 2. Materials and Methods

### 2.1. Strains and Culture Conditions

The wild-type strain (WT) of *M. acridum*, designated as CQMa102, has been archived at the China General Microbiological Culture Collection Center (CGMCC, Beijing, China; No. 0877). Cultivation of all fungal strains was typically conducted on a diluted Sabouraud’s dextrose agar medium, specifically at one-quarter strength (referred to as 1/4 SDAY), which comprised 1% dextrose, 0.25% mycological peptone, 0.5% yeast extract, and 2% agar, with a weight-to-volume ratio, at a pH of 6.4. The medium was incubated at 28 °C for a period of 15 days. For DNA manipulation and transformation procedures, the *Escherichia coli* DH5 (TransGen Biotech, Beijing, China) was grown in Luria–Bertani (LB) broth at 37 °C, while *Agrobacterium tumefaciens* AGL-1 (TransGen Biotech, Beijing, China) was cultured at 28 °C under the same conditions.

### 2.2. Bioinformatic Analysis

The complete *MaPac2* sequence was obtained from the genome database of *M. acridum* strain CQMa102 [27]. Total RNA extraction from the fungus was performed utilizing the Ultrapure RNA Kit (CWBIO, Beijing, China). Complementary DNA (cDNA) synthesis was carried out with the PrimeScript™ RT Master Mix (TaKaRa, Dalian, China). The full-length cDNA of *MaPac2* was amplified using the primer pair *Pac2*-F and *Pac2*-R, details of which are presented in Supplementary Table S1. The PCR outcomes were cloned into the pMD19-T vector (TaKaRa, Dalian, China) and then introduced into *E. coli* DH5α for sequencing purposes. For the identification of the fungal Pac2 protein, the BLAST tool on the NCBI website (http://www.ncbi.nlm.nih.gov, accessed on 10 September 2023) was employed. A phylogenetic tree was constructed using MEGA version7.0, employing the neighbor-joining approach and validated with a bootstrap test consisting of 1000 replications. Predictions of protein domains were made accessible through the SMART online tool (http://smart.embl-heidelberg.de/, accessed on 10 September 2023).

### 2.3. Constructions of the Mutants

To elucidate the role of *MaPac2* in *M. acridum*, the gene was inactivated using the split-marker method through homologous recombination. The construction of the *MaPac2* disruption vectors is detailed below. Fungal genomic DNA extraction was performed as previously described [28]. For the creation of the disruption vectors pK2-SM-L-*MaPac2* and pK2-SM-R-*MaPac2*, flanking sequences of 1.4 kb (left border) and 1.0 kb (right border) surrounding the *MaPac2* gene were amplified. The left border sequence was integrated into the *HindIII/XbaI*-digested pK2-SM-F vector, resulting in pK2-SM-L-*MaPac2* [29]. Similarly, the right border was incorporated into the *EcoRV/EcoRI*-digested pK2-SM-R vector to form pK2-SM-R-*MaPac2* [29]. For the *MaPac2* complementation vector pK2-sur-*MaPac2*::EGFP, a 3.7 kb fragment encompassing the 1.3-kb *MaPac2* coding sequence and a 2.4 kb promoter region was PCR-amplified from *M. acridum* wild-type genomic DNA. This fragment was then cloned into the *HindIII/BamHI*-digested pK2-sur-egfp vector, which was modified by inserting an egfp gene into the *BamHI* and *EcoRV* sites of the pK2-sur vector, yielding pK2-sur-*MaPac2*::EGFP. The recombinant plasmids were then introduced into AGL1 for fungal transformation. Δ*MaPac2* knockout transformants, which were strains with the targeted gene inactivated, were selected on Czapek-dox agar (CZA) medium supplemented with 500 μg/mL glufosinate-ammonium (Sigma, St. Louis, MO, USA), while complementary (CP) transformants were identified on CZA medium with 20 μg/mL chlorimuron ethyl (Sigma, Bellefonte, PA, USA), followed by PCR screening [29]. Reverse transcription-quantitative PCR (RT-qPCR) was conducted to verify all fungal transformants. Primers for screening and validation of fungal transformants are detailed in Supplementary Table S1.

### 2.4. Phenotypic Analyses

Germination of conidia on 1/4 SDAY was evaluated according to the methods as described previously [29]. In summary, 100 µL of fungal strain conidial suspensions at a concentration of 1 × 10^7^ conidia/mL was evenly distributed onto 1/4 SDAY medium and incubated at 28 °C. The percentage of germinated conidia was determined at 2 h intervals under microscopic examination until complete germination was observed. The yield of conidia was measured as described previously [29]. Specifically, 2 μL of conidial suspensions at a density of 1 × 10^6^ conidia/mL was placed onto 12-well plates containing 2 mL of 1/4 SDAY medium per well. The number of conidia produced was recorded at two-day intervals starting from day three.

To assess strain vulnerability to heat shock, 100 µL aliquots of conidial suspensions at a density of 1 × 10^7^ conidia/mL was subjected to 45 °C for durations of 0, 2, 4, 6, or 8 h. Post-treatment, these suspensions were spread onto 1/4 SDAY agar. In the case of UV-B resistance evaluations, 100 µL of the same conidial concentration from various strains was evenly distributed on 1/4 SDAY plates and exposed to UV-B radiation (Aoyi Instruments, Shanghai, China) at an intensity of 1350 mW/m^2^ for 1, 2, 3, or 4 h. Following these treatments, the plates were incubated at 28 °C for 20 h to determine the germination rates of the conidia. Each treatment was conducted in triplicate for consistency.

To assess the impact of various chemicals on the integrity of the fungal cell wall, we applied 2 µL of a conidial suspension at a concentration of 1 × 10^7^ conidia/mL onto both standard 1/4 SDAY agar and modified 1/4 SDAY agar containing different stressors: 140 μg/mL calcofluor white (CFW), 0.01% sodium dodecyl sulfate (SDS), 500 μg/mL Congo red, 6 mM hydrogen peroxide (H_2_O_2_), or 1 M sorbitol. Following a 6-day incubation period, photographs of the fungal colonies were taken, and the inhibitory effects of these chemicals on fungal growth were evaluated. The relative growth inhibition (RGI) was determined using the formula: [(average control colony diameter–average stressed colony diameter)/average control colony diameter] × 100. This calculation provided a percentage value reflecting the growth suppression caused by each chemical. Each experiment was repeated three times to ensure the accuracy of the results [30].

### 2.5. Insect Bioassays

Virulence assessments of the fungal strain were conducted using both topical inoculation and injection techniques, with fifth-instar nymphs of *Locusta migratoria manilensis* serving as the subjects. The purpose of these assessments was to determine the potency of the fungal strain in causing mortality. In the topical inoculation approach, a 5 µL droplet of conidial suspension at a density of 1 × 10^7^ conidia/mL was applied to the insects’ head-thorax boundary for the experimental group, whereas the control group received an equivalent volume of pure paraffin oil. For the injection method, 5 µL of conidial suspensions, prepared in sterile double-distilled water (ddH_2_O) at a concentration of 1 × 10^8^ conidia/mL, was injected into the hemolymph of the locusts; the control group was injected with ddH_2_O alone. Each treatment consisted of 30 locusts and was replicated thrice under consistent environmental conditions: a constant temperature of 28 °C, a relative humidity ranging from 45% to 70%, and a photoperiod of 16 h light and 8 h darkness. Mortality rates were documented every 12 h until the complete demise of the test insects, and the median lethal time (LT_50_) was determined to assess the potency of the fungal strain.

### 2.6. Cuticle Penetration Assays and Appressorium Formation on Locust Wings

A conidial suspension at a density of 1 × 10^7^ conidia per milliliter, prepared with 0.05% Tween-80, was used for the experiment. Sterile hind wings of locusts were placed on 1/4 SDAY solid medium and inoculated with 2 µL of the conidial suspension from various fungal strains. After a 2-day incubation period at 28 °C, the wings were removed to continue incubation for an additional 4 days, after which images were captured and the diameters of the colonies were measured. Concurrently, a control group was cultivated on 1/4 SDAY plates for a total of 6 days.

Conidial germination and appressorium formation on locust wings were assessed using previously established methods [31]. The conidial suspensions were inoculated onto autoclaved locust wings and incubated at 28 °C for varying durations. The germination rate was recorded every 3 h starting from 0 h, while appressorium formation was counted every 3 h beginning at 14 h.

### 2.7. Fungal Growth in Locust Hemolymph In Vitro and Nodule Formation Assays

In vitro assays of fungal proliferation within locust hemolymph were conducted by introducing conidial suspensions, at a density of 1 × 10^6^ conidia/mL, in 10 µL aliquots into 500 µL of cell-free locust hemolymph. These cultures were agitated on a rotary shaker at 28 °C and a speed of 250 rpm for durations of 2 and 3 days. Quantitative real-time PCR was employed to ascertain the genomic DNA (gDNA) concentrations, indicative of fungal growth within the locust’s blood. Additionally, a 50 mL volume of TBP liquid medium was inoculated with 1 mL of a conidial suspension at a concentration of 5 × 10^6^ conidia/mL and incubated on a shaker at 28 °C to mimic fungal development in locust hemolymph [32]. The biomass accumulation was evaluated after a 3-day period.

For the injections, a suspension of conidia, prepared in sterile double-distilled water (ddH_2_O) to a density of 1 × 10^8^ conidia per milliliter, was used. Five milliliters of conidial suspension was administered through the intersegmental membrane located between the second and third segments of the locust’s abdomen. Twenty-four hours post-injection, the formation and quantity of nodules were examined and documented.

### 2.8. Fluorescent Staining of Cell Wall Components

A suspension of conidia was crafted at a density of 1 × 10^7^ conidia/mL, with 0.05% Tween-80 serving as the solvent, and then, it was washed thrice using a 0.01 M PBS buffer. For the identification of α-1,3-glucan within the fungal cell wall, IgM MOPC-104E (Sigma, St. Louis, MO, USA) was employed in conjunction with an Alexa Fluor 488 goat anti-mouse IgM antibody (Invitrogen, Carlsbad, CA, USA). The presence of β-1,3-glucan was ascertained using a β-1,3-glucan-specific antibody (Biosupplies, Parkville, Australia) complemented by an Alexa Fluor 594 goat anti-mouse IgG antibody (Invitrogen, Carlsbad, CA, USA). The components mannose and chitin were evaluated with Concanavalin A (ConA) (Vector Laboratories, Burlingame, CA, USA) and wheat germ agglutinin (WGA) (Invitrogen, Carlsbad, CA, USA), respectively. The fluorescence emitted by these cell wall constituents was examined through fluorescence microscopy, with images captured to record the observations.

### 2.9. Data Analysis

The Data Processing System was utilized to calculate the median germination time (GT_50_) for achieving 50% conidial germination, the LT_50_ for the bioassayed locusts, and the median inhibition time (IT_50_) for the inhibition of 50% conidial germination due to heat shock and UV-B radiation. The GT_50_, LT_50_, and IT_50_ were determined through Probit analysis using GraphPad Prism 8. Experimental data from three replicate trials were analyzed using one-way analysis of variance (ANOVA) within SPSS version 22.0 (IBM, Armonk, NY, USA). Following the ANOVA, Tukey’s honestly significant difference (HSD) test was applied to compare the mean values among various strains.

## 3. Results

### 3.1. Features of Pac2 in M. acridum and Generation of Its Mutant Strains

**Figure 1:**
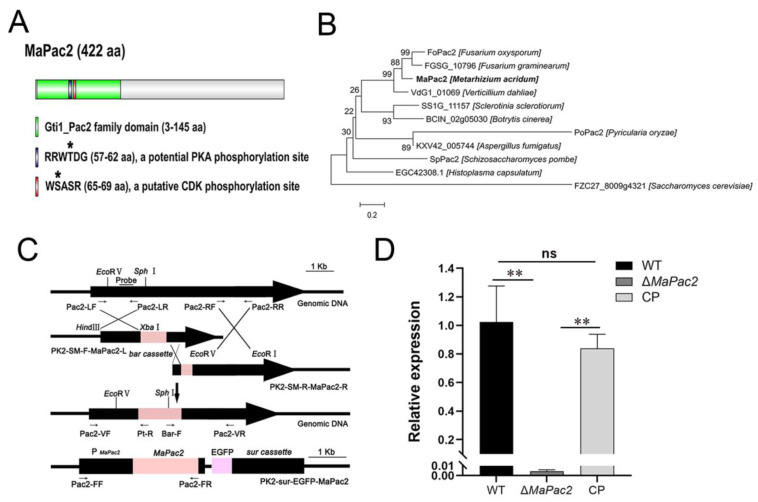
Bioinformatics analysis of *MaPac2* protein and constructions of vectors. (**A**) Domain graph of *MaPac2* proteins with DOG 1.0. Asterisks represent the putative phosphorylation sites. (**B**) Phylogenetic analysis of *MaPac2* protein with MEGA 7.0. Bold words represent the Pac2 homologous protein in *M. acridum*. (**C**) Construction of *MaPac2* deletion and complementation vectors. Black arrows indicate the positions of primers. (**D**) The relative expression levels of *MaPac2* in WT, Δ*MaPac2*, and CP strains were analyzed by RT-qPCR. WT—the wild-type strain; Δ*MaPac2*—the *MaPac2* deletion strain; CP—the *MaPac2* complemented strain. Asterisks indicate significant difference at (**) *p* < 0.01 and (ns) *p* > 0.05.

Domain analysis revealed that *MaPac2* encodes a protein consisting of 422 amino acids, featuring a conserved Gti1/Pac2 family domain (Pfam09729) across the fungal kingdom. Additionally, *MaPac2* was predicted to have a protein kinase A (PKA) phosphorylation site at the N-terminus and a potential cyclin-dependent kinase (CDK) phosphorylation site (Figure 1A). MEGA v7.0 was employed for constructing a phylogenetic tree using the neighbor-joining method, which demonstrated that *MaPac2* is highly conserved in filamentous fungi based on sequence homology (Figure 1B).

To elucidate the biological role of *MaPac2* in *M. acridum*, targeted gene disruption mutants and complemented strains were developed as described in the Materials and Methods section. The gene disruption involved replacing genomic target regions with a 900 bp phosphinothricin resistance cassette (bar) (Figure 1C). Complemented strains were created through ectopic insertion with promoter regions (Figure 1C). Preliminary screening of potential transformants was conducted using PCR. Subsequent verification was carried out using RT-qPCR. As a result, the expression level of *MaPac2* in the Δ*MaPac2* strain was significantly decreased compared with those in the WT and CP strains (*p* < 0.01; Figure 1D).

### 3.2. Deletions of MaPac2 Promoted the Conidial Germination and Decreased the Conidial Yield

**Figure 2:**
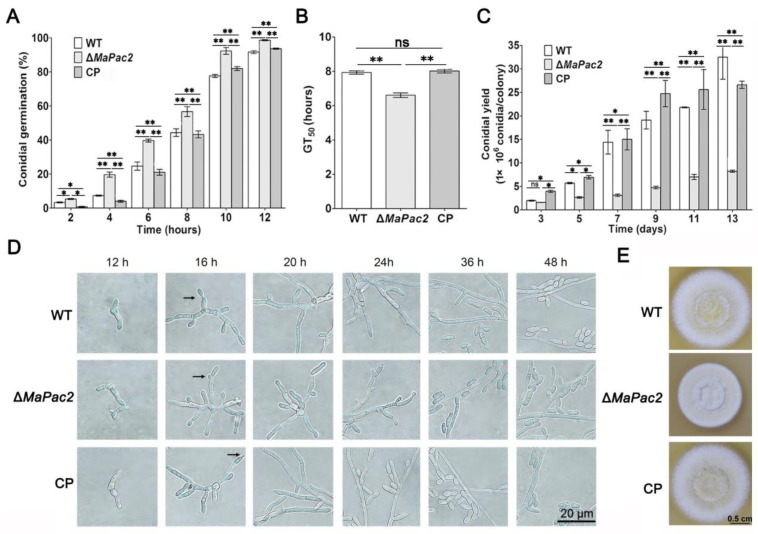
Germination assays and conidiation assays of fungal strains. (**A**) Germination rates of fungal strains incubated for 2, 4, 6, 8, 10, and 12 h on 1/4 SDAY medium. (**B**) GT_50_s of fungal strains. Error bars indicate the standard deviations. WT—the wild type; Δ*MaPac2*—the *MaPac2* deletion mutant; CP—the *MaPac2* complemented transformant. (**C**) Conidia of each strain on 1/4 SDAY media at 28 °C for 3 d, 5 d, 7 d, 9 d, 11 d, and 13 d. (**D**) Conidiation pattern of Δ*MaPac2* and complementation strains grown on SYA media. (**E**) Colonies of each strain grown on 1/4 SDAY media at 28 °C for 6 d. WT—the wild-type strain; Δ*MaPac2*—the *MaPac2* deletion strain; CP—the *MaPac2* complemented strain. Asterisks indicate significant difference at (*) *p* < 0.05, (**) *p* < 0.01, and (ns) *p* > 0.05.

To assess the impact of *MaPac2* on the germination of conidia, we conducted germination tests on 1/4 Sabouraud Dextrose Agar (SDAY). The results demonstrated a significant increase in conidial germination in the absence of *MaPac2*. Pronounced differences in germination rates were noted between the wild-type (WT) and the Δ*MaPac2* strain at intervals of 4, 6, 8, 10, and 12 h (Figure 2A). The Δ*MaPac2* strain achieved 50% germination (GT_50_) in a markedly shorter time frame, with an average of 6.3 ± 0.1 h, as opposed to the WT which had 7.9 ± 0.15 h (*p* < 0.01; Figure 2B). Furthermore, we monitored the conidial production of the WT, Δ*MaPac2*, and complementation (CP) strains on 1/4 SDAY, recording observations every two days. The Δ*MaPac2* strain exhibited a significantly reduced conidial yield, with a noticeable decrease starting from day 5 and a 60% lower yield compared to the control strains by day 13 (*p* < 0.01; Figure 2C).

To explore the cause of the substantial decrease in conidia production for the Δ*MaPac2* strain in 1/4 SDAY medium, we examined the conidia production patterns of the WT, Δ*MaPac2*, and CP strains on this medium. No differences in conidial production mode were detected among the three strains, suggesting that the reduction in conidial production was not attributable to changes in the conidia production process (Figure 2D). Furthermore, when cultured on 1/4 SDAY medium for 6 days, the colony size and diameter of the Δ*MaPac2* strain were significantly smaller than those of the WT and CP strains (Figure 2E). These observations highlight the pivotal role of *MaPac2* in both conidial germination and overall fungal growth.

### 3.3. Deletion of MaPac2 Enhances Tolerances to UV-B Irradiation and Heat Shock and Increases Sensitivity to SDS, CR, NaCl, and SOR

**Figure 3:**
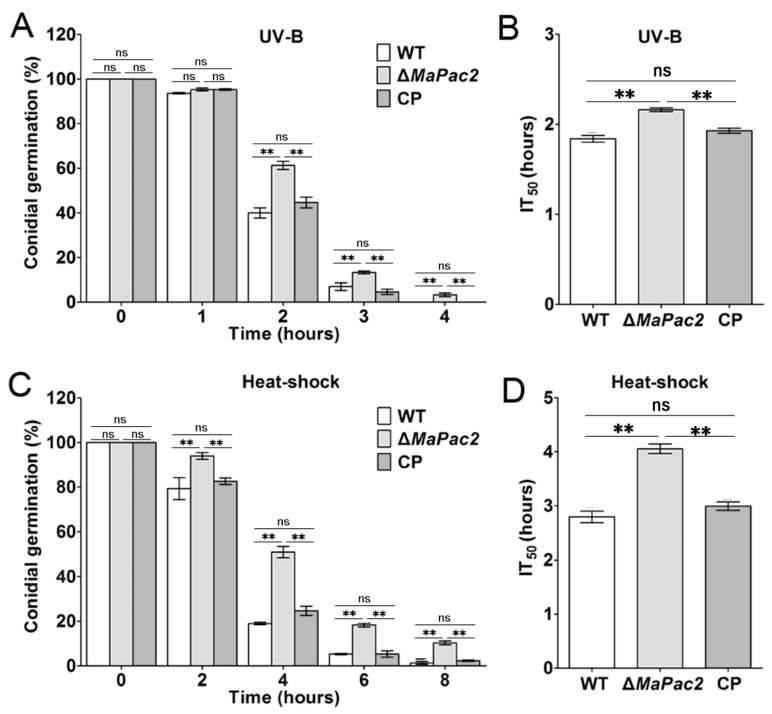
Stress tolerance assays to UV-B irradiation and wet heat of fungal strains. (**A**) Germination rates of fungal conidia treated with UV-B irradiation at 1350 mW/m^2^ for 1 h, 2 h, 3 h, and 4 h. (**B**) IT_50_s of fungal strains treated with UV-B. (**C**) Gemination rates of fungal conidia treated with wet heat at 46 °C for 3 h, 5 h, 7 h, and 9 h. (**D**) IT_50_s of fungal strains treated with wet heat. Error bars indicate the standard deviations. WT—the wild-type strain; Δ*MaPac2*—the *MaPac2* deletion strain; CP—the *MaPac2* complemented strain. Asterisks indicate significant difference at (**) *p* < 0.01 and (ns) *p* > 0.05.

In our study, we investigated the influence of the *MaPac2* gene on the heat and UV tolerance of *M. acridum*. Our research findings indicated that the Δ*MaPac2* strain displayed markedly distinct resistance to UV-B irradiation and heat stress when compared with both the wild-type (WT) and control (CP) strains. Following a 2 h exposure to UV-B, the germination rate of Δ*MaPac2* conidia (~62%) was considerably higher than those of the WT (~40%) and CP (~45%; *p* < 0.01; Figure 3A). The time required for 50% inhibition of germination (IT_50_) under irradiation was notably shorter for the WT (1.8 ± 0.1 h) and CP (1.9 ± 0.3 h) than for Δ*MaPac2* (2.2 ± 0.2 h; *p* < 0.01; Figure 3B). Similar patterns were observed in heat tolerance assessments. Upon a 4 h exposure to 46 °C, the germination rate of Δ*MaPac2* conidia (~51%) surpassed that of the WT (~19%) and CP (~25%; *p* < 0.01; Figure 3C). The IT_50_s for germination under heat stress were significantly lower for the WT (2.8 ± 0.2 h) and CP (3.0 ± 0.3 h) compared to that for Δ*MaPac2* (4.1 ± 0.1 h; *p* < 0.01; Figure 3D). Additionally, the fungal strains were cultivated on 1/4 SDAY medium supplemented with various chemicals to evaluate their stress tolerance during growth.

**Figure 4:**
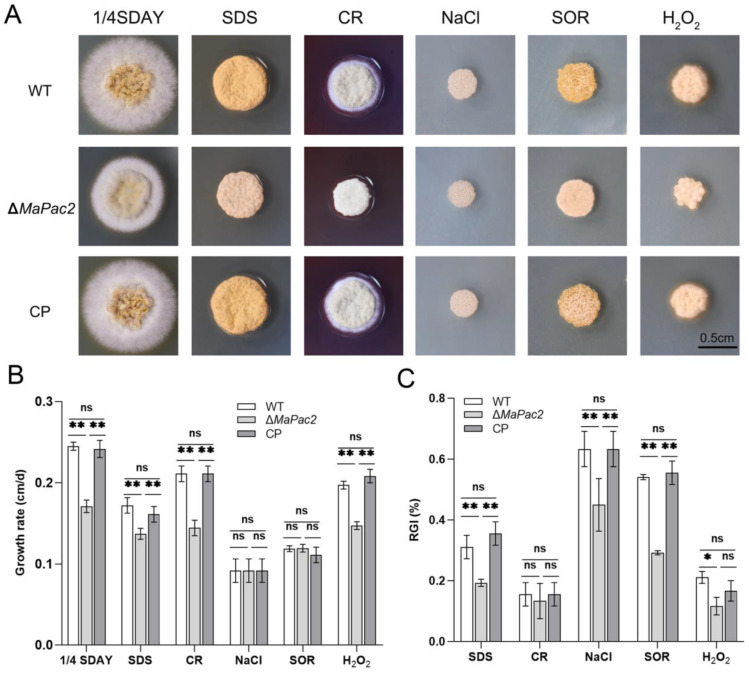
The resistance of WT, Δ*MaPac2*, and CP strains to different chemicals. (**A**) Colony growth on 1/4SDAY solid medium and 1/4 SDAY solid medium with different chemical reagents. Bar: 0.5 cm. (**B**) Relative growth rate of colony. (**C**) Relative inhibition rate of colony. All experiments were repeated three times. WT—the wild-type strain; Δ*MaPac2*—the *MaPac2* deletion strain; CP—the *MaPac2* complemented strain. Asterisks indicate significant difference at (*) *p* < 0.05, (**) *p* < 0.01, and (ns) *p* > 0.05.

Our findings revealed that the Δ*MaPac2* strain exhibited insensitivity to hyperosmotic stress induced by NaCl and SOR (Figure 4A–C). Additionally, the strain displayed reduced resistance to agent, SDS, which disrupted cell membrane (Figure 4A–C). Similarly, the Δ*MaPac2* strain showed heightened tolerance to oxidative stress conditions induced by H_2_O_2_ when compared to the WT strain (Figure 4A–C). These results underscore the significant roles of *MaPac2* in conferring multiple stress tolerance to *M. acridum*, suggesting that its modulation could be a key factor in enhancing the resilience of this entomopathogenic fungus against environmental stressors.

### 3.4. Deletion of MaPac2 Increases Fungal Virulence by Injection

**Figure 5:**
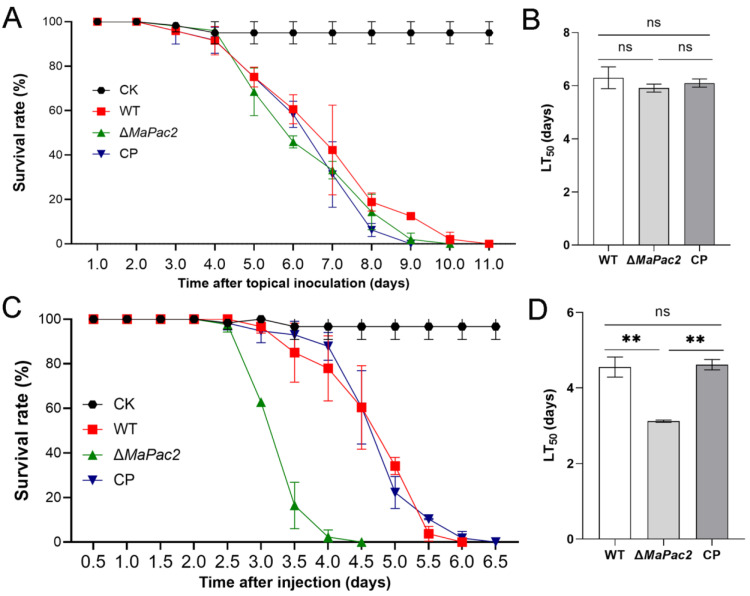
Deletion of *MaPac2* increases fungal virulence by in vivo injection. (**A**) Survival of locusts after topical application of 5 µL paraffin oil conidial suspension from WT, Δ*MaPac2*, and CP. Control insects were treated with 5 µL paraffin oil. (**B**) The LT_50_s of the Δ*MaPac2*, WT, and CP strains after topical inoculation. (**C**) Survival of locusts after injection of 5 µL ddH_2_O conidial suspension from WT, Δ*MaPac2*, and CP. Control insects were treated with 5 µL ddH_2_O. (**D**) The LT_50_s of the Δ*MaPac2*, WT, and CP strains after injection. WT—the wild-type strain; Δ*MaPac2*—the *MaPac2* deletion strain; CP—the *MaPac2* complemented strain. Asterisks indicate significant difference at (**) *p* < 0.01 and (ns) *p* > 0.05.

To assess the effect of the *MaPac2* gene on the pathogenicity of *M. acridum*, we utilized fifth-instar nymphs of *L. migratoria* manilensis for pathogenicity testing of the wild-type (WT), Δ*MaPac2*, and CP strains through both topical application and injection methods. In the topical inoculation experiments, no significant differences in virulence were detected among the Δ*MaPac2*, WT, and CP strains (Figure 5A). The LT_50_ values for these strains were 5.91 ± 0.105 days, 6.3 ± 0.2 days, and 6.10 ± 0.1 days, respectively (*p* > 0.05; Figure 5B). In contrast, the Δ*MaPac2* strain demonstrated a significant increase in virulence in injection experiments compared to the WT and CP strains (Figure 5C). The LT_50_ for the Δ*MaPac2* strain was 3.12 ± 0.02 days, which was markedly shorter than that for WT (4.55 ± 0.3 days) and CP (4.62 ± 0.2 days) (*p* < 0.01; Figure 5D).

### 3.5. Deletion of MaPac2 Reduces the Cuticle Penetration but Enhances the Colonization in Locust Hemolymph

**Figure 6:**
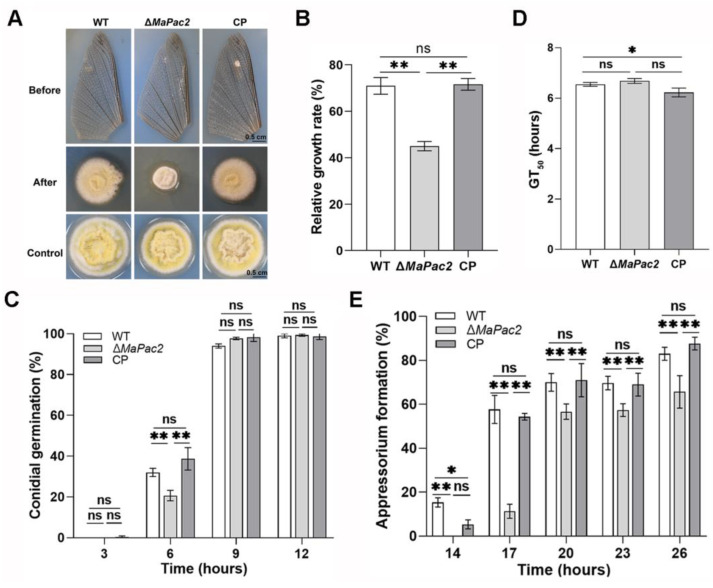
Deletion of *MaPac2* reduces fungal virulence and affects the formation of appressorium on locust wings. (**A**) Penetration assays. (**B**) Relative growth of colonies of different strains in penetration experiments. (**C**) Germination of conidia on the locust wings of fungal strains. (**D**) The GT_50S_ of different strains on locust wings. (**E**) Appressorium formation of different fungal strains on locust wings. WT—the wild-type strain; Δ*MaPac2*—the *MaPac2* deletion strain; CP—the *MaPac2* complemented strain. Asterisks indicate significant difference at (*) *p* < 0.05, (**) *p* < 0.01, and (ns) *p* > 0.05.

**Figure 7:**
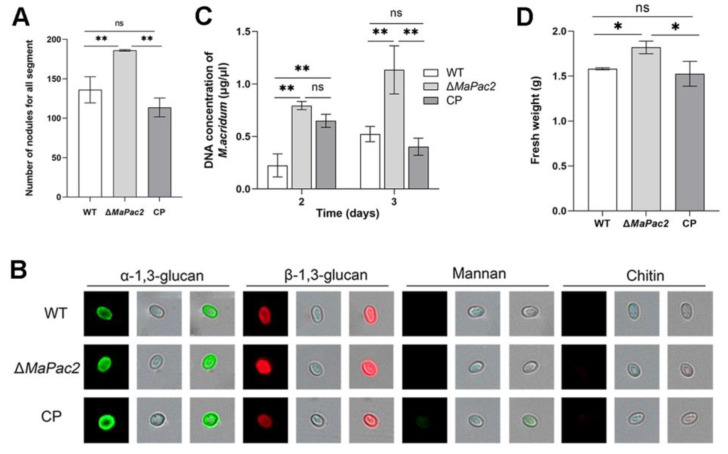
Deletion of *MaPac2* reduces the cuticle penetration but enhances the colonization in locust hemolymph. (**A**) The number of nodules after injection at 24 h on ventral of insect body walls. (**B**) Detection of conidial cell surface components with labeled lectins and antibodies. (**C**) Concentration of fungal gDNA in host hemolymph without blood cells in vitro. (**D**) Fresh weight (g) of each strain after inoculation to TBP liquid medium for 3 days. WT—the wild-type strain; Δ*MaPac2*—the *MaPac2* deletion strain; CP—the *MaPac2* complemented strain. Asterisks indicate significant difference at (*) *p* < 0.05, (**) *p* < 0.01, and (ns) *p* > 0.05.

Penetration assays of the fungal strains revealed that the colony size of the Δ*MaPac2* strain was significantly reduced compared to those of other strains (Figure 6A). Additionally, the growth rate of these colonies was considerably lower when compared with the wild-type (WT) and control (CP) strains (*p* < 0.01; Figure 6B). We also quantified germination and appressorium formation for the WT, Δ*MaPac2*, and CP strains on locust hindwings. After a 6 h incubation, the germination rate of Δ*MaPac2* was 21 ± 2.0%, which was substantially lower than those of WT (32 ± 1.6%) and CP (39 ± 4.5%) (*p* < 0.01; Figure 6C). However, by 9 and 12 h of incubation, there was no significant difference in germination rates between Δ*MaPac2* and the other strains (*p* > 0.05; Figure 6C). The time to achieve 50% germination (GT_50_) was not significantly different among the three strains (*p* > 0.05; Figure 6D). Notably, appressorium formation in Δ*MaPac2* was consistently and significantly lower. At 26 h of incubation, the rate of adherent cell formation in Δ*MaPac2* was 65.7 ± 6.0%, markedly less than those of WT (83 ± 2.5%) and CP (87.7 ± 2.4%) (*p* < 0.01; Figure 6E). These results indicate that the deletion of *MaPac2* impairs the ability of *M. acridum* to penetrate the insect cuticle. Post-hemocoel injection, the number of nodules formed within 24 h was counted. The Δ*MaPac2* strain produced a significantly higher nodule count of 186 ± 1 compared to WT (134 ± 16) and CP (114 ± 10) (*p* < 0.01; Figure 7A). Using lectins or specific antibodies to stain cell surface components, we found that the absence of *MaPac2* increased the presence of β-1,3-glucan without significantly altering α-1,3-glucan, mannose, or chitin distribution (Figure 7B). These results suggest that the loss of *MaPac2* does not enhance the fungus’s ability to avoid the host’s immune response. Furthermore, we collected 500 µL of hemocyte-depleted hemolymph from fifth-instar nymphs of L. migratoria manilensis and introduced the three fungal strains for in vitro cultivation. Quantitative PCR (qPCR) was employed to measure the absolute quantity of fungal genomic DNA from the three fungal strains within the locust hemolymph under in vitro conditions. The Δ*MaPac2* strain exhibited a higher concentration of genomic DNA than the WT strain (*p* < 0.01; Figure 7C).

Following a 3-day period of incubation, the concentration of genomic DNA in the Δ*MaPac2* strain escalated to an average of 1.13 ± 0.16 ng/μL, significantly surpassing the levels observed in the wild-type (WT) strain with 0.52 ± 0.06 ng/μL and the control (CP) with 0.40 ± 0.06 ng/μL (Figure 7C). Thereafter, we performed an experiment to mimic the proliferation of *M. acridum* in the hemolymph of locusts by employing a TBP liquid medium. The outcomes indicated a notably greater biomass accumulation in the Δ*MaPac2* strain as opposed to both the WT and CP strains (Figure 7D). Collectively, these results imply that the absence of *MaPac2* boosts the ability of M. acridum to colonize, attributed to the expedited growth rate within the locust’s hemolymph.

## 4. Discussion

The transcription factor Gti1/Pac2, which is distinctive to fungi, possesses a conserved architectural framework. Existing research results indicate that this family regulates the growth, development, conidia formation, and virulence of filamentous fungi, and the orthologs of Gti1 and Pac2 exhibit similar or distinct functions across different fungal species. In F. graminearum, the Gti1 homolog Fgp1 regulates conidiation, toxin synthesis, and pathogenicity, while the disruption of Fgp2, the Pac2 homologous gene, does not reduce the pathogenicity, and the accumulated levels of mycotoxins in ΔFgp2 are comparable to the wild-type strain [14]. In S. pombe, under conditions of incomplete starvation, Pac2-deficient cells can express stell, a gene for the key transcription factor in regulating the sexual development, and enter sexual development, while overexpression of Pac2 inhibits sexual development [8]. In the case of the rice blast fungus, M. oryzae, the elimination of the MoPac2 gene leads to a substantial decrease in the dry weight of the hyphae, and the yield of conidia is roughly double that of the wild-type strain [10]. Virulence tests have shown that the strain lacking MoPac2, denoted as ΔMoPac2, exhibits reduced pathogenicity compared to the wild-type strain. Additionally, there is an increased expression of pathogen-responsive genes in rice plants when infected with ΔMoPac2, as opposed to those infected with the wild-type strain [10]. Current research on this family mainly focuses on Gti1; therefore, it is essential to explore the role of Pac2 in other fungi. In *M. acridum*, Pac2 contributes to conidial development and growth, stress resistance, and pathogenicity.

After the deletion of the *MaPac2* gene, compared to the WT strain, the colony size was reduced, the conidial germination was accelerated, and the conidial yield was significantly decreased. We observed the conidiation pattern and found no significant differences from the wild type, suggesting that the reduction in conidial yield might be due to decreased biomass accumulation. It has been reported that Pac2 is involved in the sexual reproduction process of fission yeast. In the rice blast fungus, after knocking out MoPac2, the colony size on PDA and SDC media was slightly reduced, while conidial yield was greatly increased, approximately doubling that of the WT [10,21]. These results indicate that the deletion of Pac2 in both *M. oryzae* and *M. acridum* leads to a reduction in colony size, but the roles of Pac2 in sporulation are different between these two species.

The absence of *MaPac2* notably bolstered the conidia’s resilience to UV-B radiation and heat stress, in addition to increasing their reactivity to chemicals like SDS, NaCl, SOR, and H_2_O_2_. In the context of M. oryzae, no marked differences were observed in the suppressive effects of H_2_O_2_ and high-osmolarity environments (NaCl and SOR) between the wild strain and the ΔMoPac2 variant. This implies that MoPac2 might be inconsequential to the fungus’s stress adaptation mechanisms [10]. However, in M. acridum, the absence of *MaPac2* changes the fungal tolerances to SDS, CR, NaCl, and SOR. These research findings indicate that the role played by the same gene is not consistent across different species.

In the topical inoculation trials, the virulence of Δ*MaPac2* showed no significant change compared to that of the WT and CP strains, but its ability to penetrate insect cuticle and the rates of appressorium formation were reduced. Interestingly, when the Δ*MaPac2* was injected into insects, its virulence was significantly enhanced compared to that of the WT strain. M. acridum produces a limited amount of toxins; hence, its pathogenicity primarily depends on its proliferation within the host hemocoel [25]. When a pathogenic fungus invades an insect host, the host immune system responds through two principal strategies: (1) identifying fungal cell wall components; and (2) forming conspicuous, dark nodules within the body cavity due to the synergistic activation of prophenoloxidase and the aggregation of hemocytes [33]. Upon breaching the insect cuticle and gaining access to the hemocoel, the fungal mycelium emerges, proliferating on the nutrients present in the hemolymph while adeptly circumventing the host immune defenses [34,35]. The ability of entomopathogenic fungi to colonize within insects is influenced by two factors: the strength of the host immune response, such as the formation of nodules, and the fungal growth rate within the host. Observing nodule formation, we found that after treatment with the Δ*MaPac2* conidial suspension, the number of nodules significantly increased, indicating that the locust immune response was not weakened, but rather intensified. In addition, the growth of Δ*MaPac2* significantly increased compared to that of the WT and CP strains in the locust hemolymph in vitro. This suggests that the increase in virulence following the knockout of *MaPac2* is primarily due to the accelerated growth of *M. acridum* within the host. In *M. oryzae*, the deletion of Pac2 led to the reduction in pathogenicity was due to defects in infectious hyphae growth and the activation of plant defense responses [10]. The differences between plant pathogenic fungi and entomopathogenic fungi may be attributed to the distinct host tissue environments. Plant cells possess a cell wall structure, and plant pathogenic fungi must penetrate the plant cell wall during invasive growth in plant tissues. Even after entering the host, their growth remains restricted. In contrast, once entomopathogenic fungi enter the host insect, they can propagate and proliferate in the insect hemolymph without the need to penetrate other insect tissues [36]. For the Δ*MaPac2* strain, impaired penetration of insect cuticle and accelerated growth in host hemolymph resulted in the various effects cancelling each other out at different stages of pathogenesis, ultimately leading to no significant changes in virulence. This indicates that Pac2 has different impacts on virulence and distinct mechanisms across different fungi.

In summary, this research delves into the traits of the *MaPac2* gene within the M. acridum species and its influence on fungal spore production, stress tolerance, and pathogenicity, offering novel perspectives on the role of *MaPac2* in entomopathogenic fungi.

## Data Availability

Data are contained within the article and Supplementary Materials.

## Supplementary Materials

The following supporting information can be downloaded at: https://www.mdpi.com/article/10.3390/jof11020100/s1, Table S1: Primers used in this study.
