# Supplementary material for: MaPac2, a Transcriptional Regulator, Is Involved in Conidiation, Stress Tolerances and Pathogenicity in Metarhizium acridum
Source: J Fungi (Basel). 2025 Jan 28;11(2):100. doi: 10.3390/jof11020100 (PMC11855946; doi:10.3390/jof11020100)
Supplement: Supplementary file 1 [file jof-11-00100-s001.zip › Supporting materials20241220.docx]

MaPac2, a transcriptional regulator, is involved in conidiation, stress tolerances and pathogenicity in *Metarhizium acridum*

Hu Xiaobin^1,2,3,4#^, Li Baicheng^1,2,3,4#^, Li Yan^1,2,3,4^, Xia Yuxian^1,2,3,4^*, Jin Kai^1,2,3,4^*

1 Genetic Engineering Research Center, School of Life Sciences, Chongqing University, Chongqing 401331, PR China;

2 Chongqing Engineering Research Center for Fungal Insecticide, Chongqing 401331, PR China;

3 Key Laboratory of Gene Function and Regulation Technologies Under Chongqing Municipal Education Commission, Chongqing 401331, PR China;

4 Joint Institute of National Engineering Research Center of Microbial Pesticides, Chongqing 401331, PR China.

^#^ These authors contributed equally to this work.

* Correspondence: jinkai@cqu.edu.cn and [yuxianxia@cqu.edu.cn](mailto:yuxianxia@cqu.edu.cn)

Table S1 Primers used in this study.

| Primers | Sequence (5' to 3') | Remarks |
| --- | --- | --- |
| Pac2-F | ATGTCAAATCAATCCAACCCTC | Used for cloning the cDNA of *MaPac2* |
| Pac2-R | CTAGGAGCGCCCTAAACCATTG |  |
| Pac2-LF | GTGGAAGTTATCGTTGATTG | Used for constructing the *MaPac2*-disruption vector |
| Pac2-LR | TTGGAGTTTTGGTCGTAAGC |  |
| Pac2-RF | AGAGTTTTGACTACTACTCC |  |
| Pac2-RR | CCATACAAGGACGGAATCTA |  |
| Pac2-VF | GCCTGGAGAAGGTCTGGTAAG | Used for screening the *MaPac2*-disruption strain |
| Pt-R | CAGCCAAGCCCAAAAAGTG |  |
| Bar-F | GCTCTACACCCACCTGCT |  |
| Pac2-VR | TCCCTGCTTCGTTTCGTG |  |
| Pac2-C-F | ACAGAGTTGACCTTCCGTAC | Used for constructing the *MaPac2*-compementary vector |
| Pac2-C-R | AATACAGAATTTCTTGTCCAGCATTC |  |
| Pac2-C-VF | CTCCACCCTACTAGCGACTCTT | Used for screening the *MaPac2*-complementary strain |
| GFP-VR | CGATGCGGTTCACCAGGGTGT |  |
| Pac2-qF | TCATCTGATTTCATACTACTC | Used for detecting the expression level of *MaPac2* by RT-qPCR |
| Pac2-qR | TACTAGACTCTGGATACATC |  |
